# Effect of Three Types of Ion Beam Irradiation on Gerbera (*Gerbera hybrida*) In Vitro Shoots with Mutagenesis Efficiency

**DOI:** 10.3390/plants10071480

**Published:** 2021-07-19

**Authors:** Tomoya Hosoguchi, Yuna Uchiyama, Hinata Komazawa, Masaki Yahata, Takashi Shimokawa, Akiyoshi Tominaga

**Affiliations:** 1 Department of Agriculture, Graduate School of Integrated Science and Technology, Shizuoka University, Shizuoka 422-8529, Japan; hosoguchi.tomoya@shizuoka.ac.jp (T.H.); uchiyama.yuna@shizuoka.ac.jp (Y.U.); yahata.masaki@shizuoka.ac.jp (M.Y.); 2 Faculty of Agriculture, Shizuoka University, Shizuoka 422-8529, Japan; komazawa.hinata.18@shizuoka.ac.jp; 3 Department of Accelerator and Medical Physics, National Institutes of Quantum and Radiological Science and Technology, Chiba 263-8555, Japan; shimokawa.takashi@qst.go.jp

**Keywords:** absorbed doses, irradiation condition, line energy transfers, mutation analysis, survival rate

## Abstract

Gerbera in vitro shoots were irradiated using three types of ion beams with different line energy transfers (LETs) to investigate the effective LET and absorbed doses for mutagenesis. Furthermore, genomic mutation analyses were conducted on the obtained mutants. Survival rate analysis showed a lower lethal dose 50% (LD_50_) with ion beams with higher LETs. Trait/morphological mutations exhibited changes in the color and shape of petals and male sterility. Irradiation conditions with the highest growth change and trait/morphological mutation rates in each ion were C irradiation at 10 Gy, Ar irradiation at 5 Gy, and Fe irradiation at 5 Gy, with a range of absorbed dose of around LD_50_ to about 10 Gy lower. The highest trait/morphological mutation rate was 14.1% with Ar irradiation at 5 Gy, which was one of the criteria for ion beam irradiation of gerbera in vitro shoots. Furthermore, the genomic mutation in the flower color, petal shape, and male sterile mutants were confirmed by genotype analysis using Genotyping by Random Amplicon Sequencing-Direct technology. This is the first study to report the efficient production of gerbera mutants that could be analyzed. Our findings may lead to more efficient gerbera mutant production and analysis technology.

## 1. Introduction

Gerbera (*Gerbera hybrida*) is a perennial plant of the genus *Gerbera* of the Asteraceae family. It produces an important cut flower in the global floricultural industry that has various flower colors and shapes. The United States has a cut flower market price of 375 million US dollars and is one of the world’s largest, with gerbera production valued at 32.7 million US dollars, accounting for 8.7% of the cut flower industry [1]. Gerbera is also a popular flower in Japan, with a production value of 3.8 billion yen (equivalent to around 38 million US dollars) [2]. The Shizuoka prefecture, which is the largest production area in Japan, is responsible for 42% of Japan’s total gerbera production [2].

The most of gerbera cultivars are generated as a result of repeated hybridization between G*erbera viridifolia* Schultz-Bip from the Transvaal in South Africa and G*erbera jamesonii* Bolus ex Hook. f. [3]. Most of the gerbera cultivars currently available in the world are produced in the Netherlands by crossbreeding [4,5]. However, growth of these cultivars in Japan required heating to ≥18 °C at night in winter, and malformed flowers, such as double stems and dead flower buds, are thought to be caused by high temperatures during the day in summer. Consumers have wide preferences for flower color and flower shape and distribution varieties change rapidly. Therefore, there is a need for rapid breeding of original varieties that meet the requirements of producers and consumers.

Mutagenesis by ion beam irradiation has recently attracted attention as an alternative breeding method to crossbreeding. Ion beam technology was developed in Japan and is characterized by high mutagenesis efficiency, even at low doses, with minimal adverse effects on growth because it can deliver higher amounts of energy more locally than X-rays and γ-rays [6], which have been used for conventional mutagenesis [7,8]. There are many examples of mutants created by ion beam irradiation in plants, such as torenia [9,10], verbena [11,12], gentian [13], cyclamen [14], petunia [15], tricyrtis hirta [16], carnation [17,18], and Colocasia [19]. And the most common case of mutant production by ion beam irradiation is in chrysanthemum [20,21,22,23,24,25,26,27,28,29], which are in the same family as gerbera, but it has not been reported in gerbera.

Ion beams can control the absorbed dose as well as the line energy transfer (LET). Recent studies using model plants have reported that irradiation with high LET is more likely to induce large-scale mutations, such as insertion and deletions of ≥100 bp and chromosomal rearrangements; this indicates the possibility of obtaining diverse mutants by changing the LET [7,30]. However, few studies have compared the effects of different LETs on horticultural crops. In mutation breeding of highly heterogeneous horticultural crops, the mutation phenotype cannot be fixed in progeny plants by self-breeding. Therefore, mutants obtained by irradiation of cultured tissue are often propagated in culture. Moreover, mutants produced by ion beam irradiation of chrysanthemum, a member of the same family as gerbera, were obtained by irradiation of cultured tissues [8], suggesting that mutant of gerbera can also be efficiently obtained by this process.

**Figure 1:**
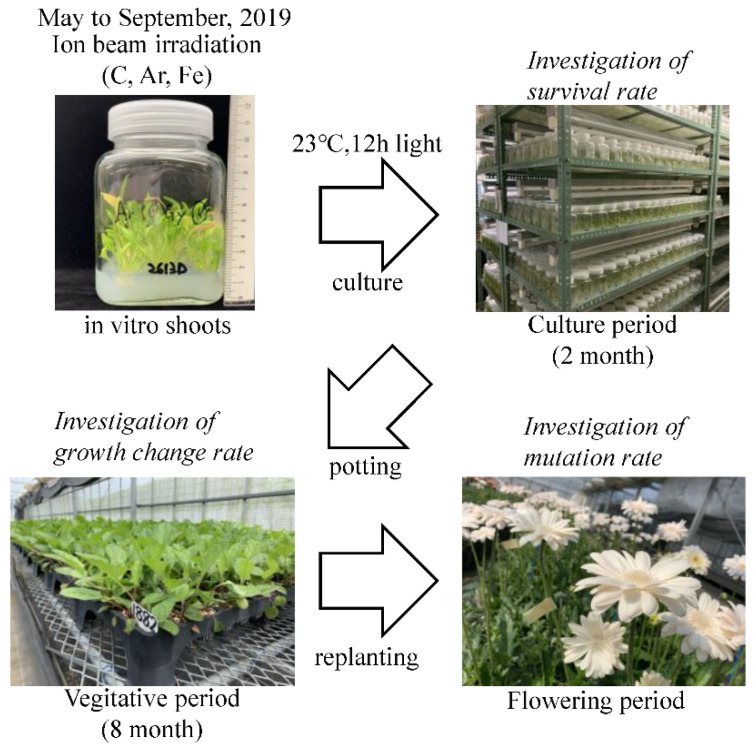
Outline of cultivation and investigation in the present study.

The present study examined irradiated cultured gerbera in vitro shoots using three different ion beams of different LETs to explore mutation breeding of gerbera. We investigated the survival, growth change, and trait/morphology mutation rates and examined the necessary ion and absorbed doses are effective for mutagenesis of gerbera (Figure 1). And Genotyping of the mutants was also performed to verify whether mutations were induced in the genomes of the gerbera mutants obtained by ion beam irradiation.

## 2. Results

### 2.1. Survival Rate

**Figure 2:**
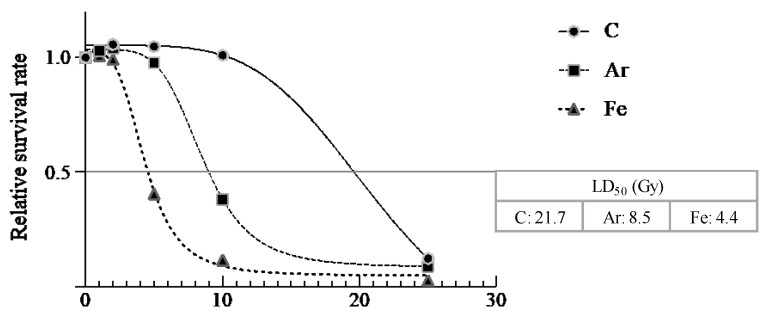
Correlation between the relative survival rate ^z^ and absorbed dose. A sigmoid curve was created using GraphPad Prism 9 and LD_50_ values were calculated. Numbers in the squares indicate the LD_50_ value. ^z^ Relative value when the survival rate of the nonirradiated plants is set to 1.

The relative survival rate was lower at the highest absorbed dose for each irradiated ion. And at all absorbed doses, the relative survival rate was highest for C irradiation, followed by Ar and Fe. The LD_50_ value was calculated by making a sigmoid curve, and the result was C irradiation at 21.7 Gy, Ar irradiation at 8.5 Gy, and Fe irradiation at 4.4 Gy (Figure 2).

### 2.2. Growth Change Rate

**Figure 3:**
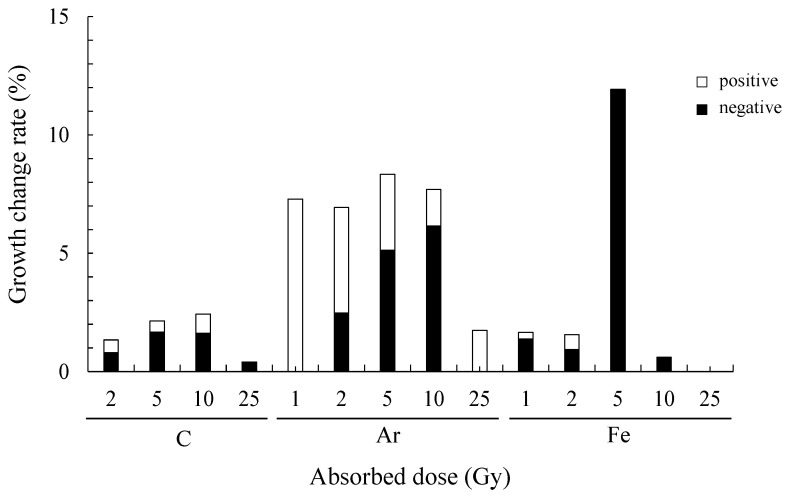
Growth change rate of gerbera obtained by ion beam irradiation.

The negative growth change rate was higher with C and Fe irradiation compared with the positive growth change rate. The positive growth change rate was higher with Ar irradiation at 1, 2, and 25 Gy (Figure 3). In plants treated with C and Ar irradiation, the negative growth change rate tended to increase as the absorbed dose increased, and then decreased when the absorbed dose exceeded a certain level for each irradiated ion (Figure 3). Following Ar irradiation, the positive growth change rate tended to decrease as the absorbed dose increased (Figure 3). The total positive and negative growth change rate in each irradiated ions were highest with C irradiation at 10 Gy (2.4%), Ar irradiation at 5 Gy (8.3%), and Fe irradiation at 5 Gy (11.9%) (Figure 3).

### 2.3. Trait/Morphological Mutation Rate

**Figure 4:**
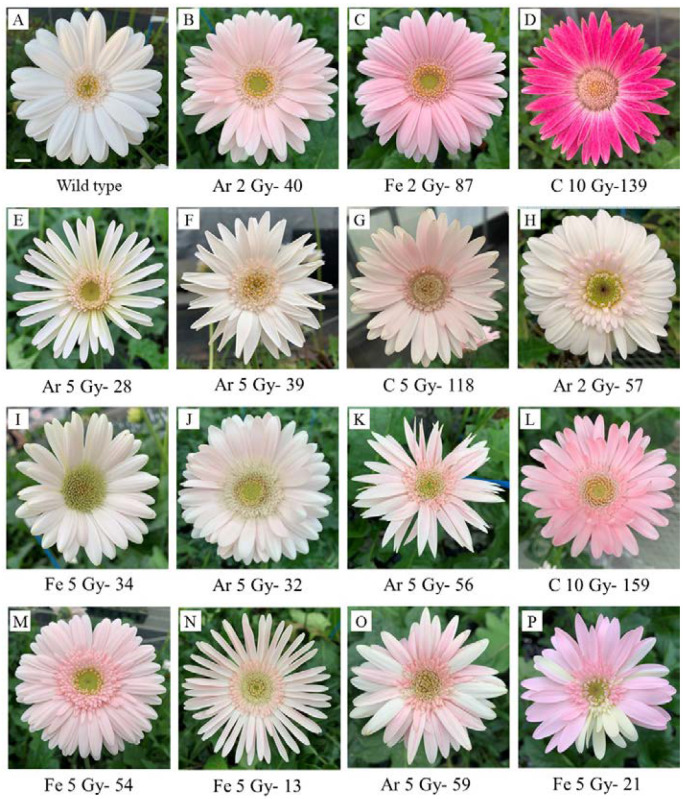
Characteristics of trait/morphological mutations obtained by ion beam irradiation. (**A**) Wild type, (**B**–**D**) flower color, (**E**) petal slender, (**F**) petal sword, (**G**) receive shape, (**H**) semidouble, (**I**,**J**) male sterile, (**K**) flower color/petal slender, (**L**) flower color/receive shape, (**M**) flower color/semidouble, (**N**) petal slender/male sterile, and (**O**,**P**) chimeric mutation. Scale bar represents 1 cm. All the numbers represent in the figure indicate line number in the irradiated plants.

During the initial growth stage, malformations, such as splitting of leaf veins, were observed in the ion beam irradiated plants; however, no malformations were observed in the newly developed leaves after irradiation. Furthermore, no plants with stable changed leaf traits and morphology were observed compared with the unirradiated plants (data not shown). Conversely, many traits and morphological variations were observed in the floral organs, such as darker red petals (Figure 4B–D), petal shape change (Figure 4E–G), semidouble flowering (Figure 4H), and male sterility (in which no pollen was seen even after flowering; Figure 4I,J) compared with the wild type flower shape of white peach petals and single flowers (Figure 4A). These were single mutations with one type of changes occurring in the same plant. Complex mutations within the same plant were also observed, including flower color/petal slender shape, flower color/petal sword shape (Figure 4K), flower color/receptive shape (Figure 4L), flower color/semidouble (Figure 4L), flower color/male sterility, and petal fineness/male sterility (Figure 4N) were confirmed. In addition, chimeric mutations (Figure 4O,P), in which petal colors were sparsely present in one inflorescence, were observed following Ar and Fe irradiation.

For a single mutation, a total of 34 plants (1.4% of the total number of irradiated plants) were obtained (Table 1). Although the degree of petal coloring was different, flower color mutants were obtained with all ion beam irradiations, and 21 plants (0.9% of the total number of irradiated plants) were obtained (Table 1). The petal shape change occurred in four patterns: slender shape, sword shape, receptive shape, and semidouble.

A total of three petal slender and petal sword shape mutants were obtained using Ar irradiation, and four petal receptive shape mutants were obtained using C and Fe irradiation. One semidouble flower mutant with change from tubular flower to ligulate flower were obtained with Ar irradiation. In addition, a total of five male sterile plants were obtained using Ar and Fe irradiation. These traits and morphological mutations were stably maintained in the later flower buds.

In terms of complex mutations, many mutations appeared at the same time as the flower color change, and a total of seven plants (0.3% of the total number of irradiated plants) were obtained (Table 1). Among these, two plants with flower color/petal slender shape mutations were obtained with C and Fe irradiation, one plant with a flower color/receptive shape mutation was obtained with C irradiation, two plants with flower color/semidouble mutations were obtained with Ar and Fe irradiation, and one plant with a flower color/male sterility mutation was obtained with Ar irradiation. One plant with petal slender/male sterility was obtained following Fe irradiation. Moreover, Ar and Fe irradiation produced three chimeric mutants with sparsely colored petals in a single inflorescence. These complex and chimeric mutations were not stable, and the presence or absence of the mutant phenotype varied in each subsequent flower bud.

**Figure 5:**
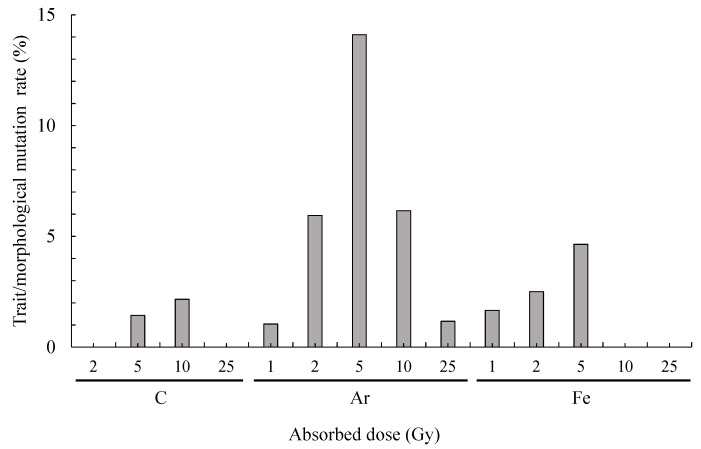
Trait/morphological mutation rate obtained by ion beam irradiation.

The total number of trait/morphological mutants was seven following C irradiation, 23 following Ar irradiation, and 14 following Fe irradiation (Table 1). The trait/morphological mutation rate increased as the absorbed dose increased and tended to decrease when the absorbed dose exceeded a certain level for each irradiated ion and was highest with C irradiation at 10 Gy (2.2%), Ar irradiation at 5 Gy (14.1%), and Fe irradiation at 5 Gy (4.6%) (Figure 5).

### 2.4. Genotype Analysis of Gerbera Mutants Obtained by Ion Beam Irradiation

Genotype analysis using GRAS-Di technology was performed to confirm whether the mutations were induced in the genome of the trait/morphological mutants of flower color Fe 2 Gy- 87, petal slender Ar 5 Gy- 29, and male sterile mutant Fe 5 Gy- 34 (Figure 4). The number of sequenced bases was 534 Mbp in the wild type, 541 Mbp in the flower color mutant, 542 Mbp in the petal slender mutant, and 510 Mbp in the male sterile mutant (Table 2). The number of genomic markers obtained was analyzed by comparing the wild type and mutants with the highly reliable base sequence, and 1 marker for flower color mutants, 6 markers for petal slender mutants, and 31 markers for male sterile mutants were identified (Table 2).

## 3. Discussion

In the present study, cultured gerbera in vitro shoots were irradiated with ion beams with different LETs to investigate the effective irradiation condition for mutation.

The survival rate of gerbera in vitro shoots irradiated with ion beams tended to decrease as the absorbed dose increased (Figure 2). The LD_50_ was 21.7 Gy for C irradiation, 8.5 Gy for Ar irradiation, and 4.4 Gy for Fe irradiation (Figure 2). These findings suggest that ion beams with higher LETs have a greater effect on the survival rate at lower absorbed doses. The irradiation conditions that resulted in the highest growth change rate and trait/morphological mutation rates were 10 Gy for C irradiation, 5 Gy for Ar irradiation, and 5 Gy for F irradiation (Figure 3 and Figure 5). These results suggest that a range of absorbed dose of around LD_50_ to about 10 Gy lower is one of the criteria for efficiently obtaining growth-changed and trait/morphology mutants.

The negative growth change rate observed following C and Ar irradiation increased with the absorbed dose and tended to decrease above a certain absorbed dose (Figure 3). It is common for growth to be negatively regulated by radiation damage in irradiated plants [8,31]. In the present study, the negative growth rate was higher than the positive growth rate in most of the irradiated areas, suggesting that radiation damage was a factor. In addition, ion beam irradiation of *Arabidopsis thaliana* induced stunted growth morphology due to large-scale genomic deletions [7,30]. Genomic deletions could be a factor in the negative growth changes were seen in the present study.

In contrast, the positive growth change rate was higher than negative growth change rate following Ar irradiation at 1, 2, and 25 Gy, and tended to decrease with increasing absorbed dose (Figure 3). C and Fe irradiation also resulted in positive growth changes, although to a lesser extent. It is already known that low doses of radiation has the stimulating effect on plants and animals (radiation hormesis) [32,33]. And a recent study reported that irradiation of *Arabidopsis* dry seeds with C-ion beams suppressed the generation of reactive oxygen species (ROS) under low temperature conditions and promoted plant growth [34]. The positive growth changes obtained in present study may also have been induced by these phenomena and should be investigated in detail in the future. In addition, mutation of cell division-related genes, such as *DA1*, promotes growth in *Arabidopsis* and *Brassica napus* [35,36], suggesting that DNA mutation may be a factor for positive growth changes. At this stage, direct radiation effects and mutational effects are both expected to appear. Since it is not possible to evaluate which is the cause at this point, it is necessary to evaluate this in the next generation.

Single mutations of trait/morphological mutants include petal color change (Figure 4B–D), petal type change (Figure 4E–G), semidouble flowering (Figure 4H), and male sterility (Figure 4I,J), and it was confirmed stably even in late flower buds.

Flower color mutations were the most frequently observed single mutations due to ion beam irradiation (Table 1). Flower color change from white to yellow by C and He irradiation was obtained in chrysanthemum due to loss of function of the carotenoid-degrading gene, *CmCCD4* [37]. There was also a flower color change from purple to white in chrysanthemum following C irradiation [24], suggesting a mutation in the anthocyanin biosynthesis gene or transcriptional factor regulation [38]. However, the flower color mutation was obtained in the present study resulted in a change from the white/pink color of the wild type to a dark pink color, and there were no cases of flower color change. Expression of genes involved in anthocyanin biosynthesis may be increased as an expected molecular mechanism, and a detailed analysis to identify the causative gene is required.

Flower shape mutations, such as petal slender shape, petal sword shape, petal receiving shape, and semidouble mutants, were also achieved (Figure 4E–H). These flower shaped mutations have not been reported in chrysanthemum, and are considered gerbera-specific mutations (Figure 4E–H).

Furthermore, male sterile mutants of gerbera were obtained as flower trait mutations (Figure 4I,J). A male sterile mutant was previously obtained by N irradiation in verbena [11,12], which did not form pollen, thus improving the longevity of the cut flowers and making it easier to use as a seed parent when crossbreeding. The same advantage was considered for male sterile mutants of gerbera (Figure 4I,J).

C, Ar, and Fe irradiation resulted in 7, 23, and 14 trait/morphology mutants, respectively. The total trait/morphology mutation rate was 1.9% (44 out of 2363 plants of the total number of irradiated plants), and the highest trait/morphological mutation rate was 14.1% with Ar irradiation at 5 Gy (Figure 5). Previous reports have shown that the mutation rate of flower color due to C irradiation (LET: 86 keV μm^−1^) on chrysanthemum petal is 4.5~11.9% [21], and was 0.4~6.4% [27] and 14.7% [25] when C irradiation (LET: 23 keV μm^−1^) was applied to the ears of chrysanthemum. Moreover, the mutation rate of flower color was 1.7~3.5% when leaves and flowers of chrysanthemum were irradiated with Ar ion beams (LET: 93 ke µm^−1^) [22]. Furthermore, as a result of C irradiation (LET: 23 keV μm^−1^) with the ears of 30 varieties of chrysanthemum, differences between varieties were observed, and a flower color mutation rate was 0% to 57.1% [24]. Based on the above previous study, the trait/morphology mutation rates in the present study were similar or slightly lower, suggesting that ion beam irradiation is an effective technique for mutation breeding in gerbera.

Although there are cases of gerbera mutants produced by γ-rays, it is highly chimeric, and it is reported that 97% of the mutants obtained even after two micropropagations after irradiation were chimeric plants [39]. In this study, the frequency of obtaining chimeric mutants is low, and chimericity was not a problem even in the same family of chrysanthemums [20,22,24]. In addition, γ-rays have a low LET and induce small deletions or point mutations in many regions of the whole genome, so it is known that mutant traits are not stable due to many mutations other than the target trait. On the other hand, it has been reported in Arabidopsis that ion beams can induce in/del of more than 100 bp and chromosomal rearrangement in less abundant regions of whole DNA due to their higher LET than γ-rays, and it is thought that it is easier to obtain mutants in which only the target trait is mutated [7,30]. Based on the above, ion beam irradiation for gerbera mutation will be useful as basic knowledge for the future production of gerbera mutants.

In addition, genotype analysis of male sterile mutants using the GRAS-Di method identified 1 marker for flower color mutant, 6 markers for petal slender mutant, and 31 markers for male sterile mutant that showed different amplification patterns were compared with the wild type (Table 2). Since GRAS-Di method does not amplify the whole genome, it is possible that more genomic mutations are induced, but it was demonstrated that genomic mutations were induced in the mutant. Although genome analysis of gerbera is difficult due to the lack of a genome reference sequence, the GRAS-Di method has proven to be an efficient method for genome sequence comparison. Future studies using molecular breeding and functional analysis using mutant-specific DNA markers are expected. Recently, whole-genome analysis using the next-generation genome sequencer is a useful technology that has come to be widely used in plants [40,41,42,43]. In this study as well, whole-genome analysis of trait/morphological mutants obtained by ion beam irradiation may clarify the specific types of genomic mutations, such as frequency of SNPs and In-Del per kb, and provide academically useful findings. However, gerbera is highly heterogeneous and has no genomic reference sequence information. Therefore, it is necessary to create a high-resolution map of the gerbera genome in order to analyze the genome of the obtained mutant. This is an issue for the future.

Furthermore, in recent years, genome editing technology has become widespread as a very useful technology for plant mutation [44,45,46,47,48,49]. However, due to the lack of genome reference information and knowledge of genes related to the target phenotype for Gerbera, it is currently difficult to mutate only specific genes, which create the expected phenotype. In addition, genome editing requires more than one year for transformation, and furthermore, backcrossing is currently required to remove selection marker genes and vector genes, which takes several years or longer. Although random mutagenesis by ion beam cannot induce targeted mutations, it can induce various mutations such as flower shape, male sterility as well as flower color even in the absence of genomic information. Furthermore, present study can produce mutants in less than one year. It is hoped that this research will reveal genes related to flower color and morphology, which will pave the way for the application of genome editing to introduce mutations.

In summary, we demonstrated for the first time that gerbera mutants can be efficiently produced by ion beam irradiation of in vitro shoots. In addition, the highest growth change and trait/morphological mutation rates were obtained using Ar irradiation at 5 Gy and were considered one of the criteria for ion beam irradiation of gerbera in vitro shoots. Furthermore, the genotype of the obtained mutants can be analyzed, and DNA marker selective breeding and molecular biological analysis of mutant genome is expected to be possible. Based on the present study, the establishment of more efficient techniques for the production and analysis of gerbera mutants, such as the expansion of irradiated cultivars based on the present study, is expected.

## 4. Materials and Methods

### 4.1. Plant Material

Gerbera “Opal” in vitro shoots (Greentech Co., Ltd., Shizuoka, Japan) in glass bottles (ca. 9 × 9 × 12 cm) were used in the present study. Ion beam irradiation was performed using a Heavy Ion Medial Accelerator in Chiba (HIMAC) at the National Institutes of Quantum and Radiological Science and Technology (Chiba, Japan). Irradiation was performed using a plateau region with a 10-cm diameter uniform-field of three ion beams (Carbon, Argon, and Iron) with different LETs (Table 3).

### 4.2. Procedure

A schematic of the procedure used in the present study is shown in Figure 1 and the number of plants irradiated with ion beams is shown in Table 4. From May to September 2019, in vitro shoots were irradiated after 4 months of cultivation using ion beams and then cultured for 2 months at 23 °C with a 12-h daylength and potted up and grown in an unheated greenhouse. Cultivation management was entrusted to Greentech Co. Although the exact details cannot be disclosed, the cultivation management was performed as previously described by Kako [50].

### 4.3. Survival Rate

Among the in vitro shoots irradiated with ion beams, those that were able to be potted without dying after about 2 months of incubation at 23 °C and 12 h light condition were defined as “survival plants”, and the ratio of the number of survival plants to irradiated plants was calculated as the survival rate. Since the timing of irradiation differed among the ion, the relative value of the survival rate of each irradiated ion to that of the nonirradiated area was calculated as the relative survival rate. The absorbed dose at which the relative survival rate became <50% was defined as the 50% lethal dose (LD_50_). To evaluate the dose response, curve fitting and LD_50_ calculation were performed by using GraphPad Prism 9.

### 4.4. Growth Change Rate

After potting, the number of leaves and maximum leaf length were measured in all plants. We visually selected the largest leaf among the plants and defined its length as the maximum leaf length. We also defined plants with leaf number and maximum leaf length less than the minimum values as negative growth change plants and plants with leaf number and maximum leaf length greater than the maximum values as positive growth change plants relative to the wild type without irradiation. The ratio of positive growth change plants to the number of irradiated plants was calculated as the positive growth change rate and the ratio of negative growth change to the number of to irradiated plants was calculated as the negative growth change rate. These values were measured 153 days after irradiation using a C ion beam, 145 days after irradiation using an Ar ion beam, and 115 days after irradiation with an Fe ion beam, which is the time of the number of leaves spread in the non-irradiation about 6.

### 4.5. Trait/Morphological Mutation Rate

The traits and morphology of the flowers were visually investigated during flowering, and plants with traits and morphologies that differed from those in the nonirradiated wild type group were defined as trait/morphology mutant plants. The ratio of the number of trait/morphology mutant plants to irradiated plants was calculated as the trait/morphology mutation rate.

### 4.6. Genotype Analysis of Gerbera Mutants Obtained by Ion Beam Irradiation

Genotype analysis using next-generation genome sequencers was performed using confirm whether mutations were induced in the genomes of the mutants. Leaves were sampled from mutants and stored at −20 °C. The sampled leaves were frozen in liquid nitrogen and ground, DNA was extracted using NucleoSpin Plant II (Takara Bio Inc., Shiga, Japan), and the yield was confirmed to be >600 ng using a biospectrometer (Eppendorf Inc., Tokyo, Japan). We confirmed that there was no degradation of DNA by electrophoresis using 2% agarose. The obtained DNA was used as a template and the entire genome was amplified and sequenced using Genotyping by Random Amplicon Sequencing-Direct (GRAS-Di) technology using a next-generation genome sequencer (Illumina, Eurofins Genomics Inc., Tokyo, Japan). In contrast to conventional genotyping by sequencing, GRAS-Di technology has fewer biased analysis regions, genome-uniform genotyping, and genomic mutation detection and marketization, even in species without reference sequences such as gerbera [51,52]. The sequences obtained from the wild type and mutant were compared and sequences that differing and determined to be highly accurate were selected to determine DNA markers specific to the mutant.

## Figures and Tables

**Table 1:** Number of mutants with trait/morphological mutations obtained by ion beam irradiation with 2363 plants.

Classification of Mutant Phenotypes		Irradiation treatment														
		C				Ar					Fe					Total
		2	5	10	25	1	2	5	10	25	1	2	5	10	25	
Single mutation	Flower color	0	1	2	0	1	4	5	1	1	2	2	2	0	0	21
	Petal slender	0	0	0	0	0	0	2	0	0	0	0	0	0	0	2
	Petal sword	0	0	0	0	0	0	1	0	0	0	0	0	0	0	1
	Receptive shape	0	2	0	0	0	0	0	0	0	0	1	1	0	0	4
	Semidouble	0	0	0	0	0	1	0	0	0	0	0	0	0	0	1
	Male sterile	0	0	0	0	0	0	2	2	0	0	0	1	0	0	5
Complex mutation	Flower color/Petal slender	0	0	1	0	0	0	0	0	0	0	1	0	0	0	2
	Flower color/Receptive shape	0	0	1	0	0	0	0	0	0	0	0	0	0	0	1
	Flower color/Semidouble	0	0	0	0	0	1	0	0	0	0	0	1	0	0	2
	Flower color/Male sterile	0	0	0	0	0	0	0	1	0	0	0	0	0	0	1
	Petal slender/Male sterile	0	0	0	0	0	0	0	0	0	0	0	1	0	0	1
Chimeric mutation		0	0	0	0	0	0	1	0	0	1	0	1	0	0	3
Total		0	3	4	0	1	6	11	4	1	3	4	7	0	0	44

**Table 2:** Summary of GRAS-Di analysis results for the mutants obtained by ion beam irradiation.

	Trait	Line Name	No. of Read	No. of Seqenced Bases (Mbp)	%Q30 ^z^	No. of Mutant-Specific Markers ^y^
Wild type	Normal	0 Gy	5,289,248	534	88.6	-
Mutant	Flower color	Fe 2 Gy- 87	5,355,904	541	89.5	1
	Petal slender	Ar 5 Gy- 29	5,364,690	542	89.2	6
	Male sterile	Fe 5 Gy- 34	5,045,048	510	88.9	31

^z^ Percentage of bases estimated to have base reliability of 99.9% or higher. ^y^ Amplification products with different sequences in the wild type and mutant.

**Table 3:** Outline of ion beam irradiation.

Elements	Ion Notation	Energy	LET
		(MeV·µ^−1^)	(keV·µm^−1^)
Carbon	^12^C^6+^	290	14
Argon	^40^Ar^18+^	500	89
Iron	^56^Fe^26+^	500	185

**Table 4:** Number of gerbera in vitro shoots irradiated with ion beams.

Ion	Absorbed Dose (Gy)	Number of Irradiated Plants	Number of Surviving Plants
C	0	201	201
	2	187	184
	5	210	205
	10	185	174
	25	123	14
Ar	0	63	63
	1	96	92
	2	101	98
	5	78	71
	10	65	23
	25	86	7
Fe	0	154	122
	1	181	171
	2	160	146
	5	151	53
	10	166	19
	25	156	4

## Data Availability

Not applicable.
